# Identification of Individual Zebrafish (*Danio rerio*): A Refined Protocol for VIE Tagging Whilst Considering Animal Welfare and the Principles of the 3Rs

**DOI:** 10.3390/ani11030616

**Published:** 2021-02-26

**Authors:** Anita Rácz, Brooke Allan, Toni Dwyer, Davide Thambithurai, Amélie Crespel, Shaun S. Killen

**Affiliations:** 1 Institute of Biodiversity, Animal Health and Comparative Medicine, College of Medical, Veterinary and Life Sciences, Graham Kerr Building, University of Glasgow, Glasgow G12 8QQ, UK; brookeann2015@outlook.com (B.A.); Toni.Dwyer@glasgow.ac.uk (T.D.); d.thambithurai.1@research.gla.ac.uk (D.T.); Amelie.Crespel@glasgow.ac.uk (A.C.); shaun.killen@gmail.com (S.S.K.); 2 Department of Genetics, ELTE Eötvös Loránd University, Pázmány P.s. 1/C, H-1117 Budapest, Hungary; 3 Department of Biology, Natura Building, University of Turku, 20500 Turku, Finland

**Keywords:** VIE, tagging, marking, fish, zebrafish, *Danio rerio*, fish welfare, Three Rs, 3Rs, analgesia

## Abstract

**Simple Summary:**

In aquatic ecology research studies commonly employ a tagging technique named visible implant elastomer (VIE). Despite existing widespread guidance on the use of this marking technique on fish, there is still a lack of information regarding efficiency in small fishes, as well as its impact on fish welfare. The current paper highlights important animal welfare issues and introduces a newly improved VIE tagging protocol, presenting information on individual survival rate, tag retention, and use of different elastomer colours, quantified in different populations and age groups of zebrafish. Specifically, we compare a previously used tagging method with a newly improved protocol which places particular emphasis to the Three Rs, helping to refine this scientific procedure. The shared detailed protocol and information will be beneficial to the zebrafish research community and beyond.

**Abstract:**

In aquatic ecology, studies have commonly employed a tagging technique known as visible implant elastomer (VIE). This method has not been widely adopted by the zebrafish research community and also lacks refinement with regard to animal welfare. The current paper introduces a new VIE tagging protocol, with the aim of improving existing tagging techniques by placing particular emphasis on the Three Rs. To improve animal welfare and fish survival, we added the use of an analgesic compound (lidocaine) through the marking procedure, followed by after-treatment with antiseptics (melaleuca, aloe vera, and PVP-I as active ingredients) to improve tissue regeneration and healing. The newly improved protocol has been quantitatively evaluated on different populations and age groups of zebrafish. This study will be useful to the scientific zebrafish community and to the wider field including biologist and aquarists, especially in consideration of animal welfare, where tagging techniques are considered as a potential noxious stimulus for fish.

## 1. Introduction

Over the past thirty years, zebrafish (*Danio rerio*) have become a popular vertebrate model organism in science [1]. Zebrafish are used to study various human diseases [2], drug developments, toxicological effects [3], and various other topics in biomedical research [4]. Recently, this model has started to become popular in behavioural, ecological, and evolutionary research as well [5,6,7,8,9,10]. In 2013, it was estimated that more than 3000 institutions in over 100 countries used zebrafish for research [11,12]. The use of this fish species in research offers several advantages: low cost of maintenance, simplicity in holding and breeding [13,14], high fecundity, small size, rapid developmental and generation times, transparent embryos, the ease of its genome manipulation to create mutant or transgenic lines, and their genetic similarity to humans [14,15,16,17]. The increased popularity of specific genetic modification techniques, such as CRISPR-*Cas9*, has also mean that zebrafish have become the organism of choice in large-scale genetic studies [12,17].

Individuals within mutant and transgenic lines used in laboratories often exhibit identical phenotypes, making visual identification of individuals impossible. Therefore, genetically different groups are typically housed separately and carefully managed to ensure proper identification (sometimes at an individual level). In many labs, multiple users are typically involved in handling and moving fish on a regular basis. This, combined with the nervous behaviour of zebrafish (i.e., jumping), can increase the chances for accidental mixing of different genetic lines. Considering these factors, it is surprising that methods to identify individuals or groups of zebrafish are still not well developed or utilised. Some facilities have begun to implement methods to aid the recognition of individual fish [18], including passive integrated transponder tags (PIT-tag) [19], p-Chips tags [20], subcutaneous dyes [21], or visible implant elastomer (VIE) [22,23,24,25] within their zebrafish colonies. However, the field is still lacking a refined, easily externally identifiable, standardized, and long-lasting marking protocol for zebrafish to use at larger scales. Visual implant elastomer (VIE) tagging is a widely used marking technique among different aquatic taxa such as fish [26,27,28,29,30,31,32,33], amphibians [34,35], cephalopods [36], and even invertebrate species [37]. Detailed information about the technique and the tested species can be found with an official tagging manual at Northwest Marine Technology, Inc website (https://www.nmt.us/wp-content/uploads/2017/11/VIE-Project-Manual-Nov-2017-1.pdf; accessed on 11 December 2017). Despite previous studies suggesting that zebrafish can be successfully tagged by following established tagging protocols [22], there is a general lack of knowledge regarding the effects of tagging procedures on the welfare of zebrafish and the degree to which specific procedural factors (e.g., the anaesthetic procedure) may generate variation among studies.

Current fish tagging protocols typically involve anaesthetisation using products such as MS-222 [38] or benzocaine. In some cases, these have been shown to be potentially deleterious for fish [39,40], leading to hypoxemia, hypercapnia, respiratory acidosis, and hyperglycaemia [41]. Tissue damage can also occur when applying the elastomer tag under the skin, which may lead to secondary infections, diseases [42,43], and can also cause immune responses in fish (increasing the granulocyte:lymphocyte ratio) [44]. These aspects of the VIE tagging raise concerns and questions, especially with regards to animal welfare and the effects of these adverse effects on scientific results.

There is often a general lack of understanding the effect of scientific procedures on animals [45,46,47], and studies on the refinement of tagging procedures are particularly sparse. The principle of the Three Rs (3Rs) is receiving growing attention as a guide for developing animal welfare in scientific research. Specifically, there is wide acceptance that the welfare of research animals (including fish) should be optimized through the 3Rs: (1) Replacement: replace animal models where it is possible or use lower taxonomic levels as replacement of mammals; (2) Reduction: reduce the number of animals used in research (could re-use same individuals in different experiments); and (3) Refinement: refine existing protocols in a way that avoids or minimises any pain, fear, distress, and lasting harm. Animals should be maintained under conditions which foster their health and wellbeing [47,48,49]. Animals with compromised welfare are likely to generate poor-quality data, thus confounding research in behaviour, physiology and immunology [47]. As such, the refinement of the existing scientific protocols and procedures is highly important and should be prioritised for the aim of avoiding any inaccurate or unreliable scientific results.

Although the capacity for fish to perceive pain has been questioned in the past [50,51], there is an accumulation of experimental evidence suggesting that, similarly to mammals, fish possess nociceptors which can detect noxious (painful) stimuli [52,53,54,55,56,57,58,59,60]. Furthermore, both the behaviour and physiology of fish are adversely affected by stimuli known to be painful to humans [52,53,54,61,62]. By extension, it is clear that further work is necessary to not only understand how such animals may perceive pain, but also how specific procedures may be adjusted to minimize responses to pain [47,61]. For example, if an experimental procedure can potentially cause tissue damage, but the aim of the study is not to detect pain, then appropriate analgesia should be applied to minimise potential animal suffering and to eliminate unwanted stress effects that may confound experimental results [45,47,57,58,61]. The use of analgesics in fish studies is limited, and most studies have tried to address how the effects of some analgesic agents may vary for different fish species and age groups [57,58,63,64,65,66,67,68,69]. Lidocaine has been shown through different behavioural and physiological data to be an effective analgesic drug in zebrafish [57,58,64,65,66]. Results have suggested that dissolving lidocaine in water prior to pain-inducing procedures can minimise the effect of potentially painful events on fish. Results have also suggested that lidocaine can be used as an after-treatment to reduce the long-term effects of procedures to speed up recovery by reducing pain. As the use of analgesic in fish studies is rather limited, further work is required to refine both protocols and how agents influence different species [69].

In addition to analgesia, the continued development of anaesthetic methods which are safe, painless, and reliable is of critical importance. There are a wide range of recommended anaesthetic substances containing a variety of compounds with different effects on fish. While the use of anaesthetics is crucial to assure fish welfare, these drugs/chemicals can also have unwanted side-effects [45,68]. Readman et al. (2017) [70] state that just focusing on the ease of application, proportion, and stability of anaesthesia is not sufficient, but that researchers should also consider the way an animal physiologically responds to anaesthesia. The use of an inadequate anaesthetic protocol can cause stress and mortality in fish colonies, compromise animal welfare, and affect scientific outcomes [45,46]. Differential responses to anaesthesia among fish species can also be problematic [71], or even among individuals of same species, depending on age, size, metabolic state, or even sex [72]. Tricaine methanesulfonate (MS-222) is the most common anaesthetic used in the zebrafish research [12]. Despite this, there is evidence that, depending on the dose and exposure, MS-222 can have adverse effects for zebrafish [39,40,72].

Herein, our aim is to provide an overview of VIE tagging in zebrafish and highlight some of the more common issues that can prove problematic. We detail an improved and standardized step-by step tagging protocol, including information on anaesthesia, analgesia, and the use of after treatment healing substances to decrease fish mortality, increase tag retention, and generally increase the welfare of the experimental animals. The protocol is useful for both the zebrafish research community and fish sciences in general, not only to improve VIE tagging, but also for a variety of other procedures that may inflict pain or tissue damage in fishes, including fin-clipping and PIT tagging [57,58].

## 2. Materials and Methods

### 2.1. Animals, Housing, and Husbandry

The fish biology group within the Institute of Biodiversity Animal Health and Comparative Medicine at University of Glasgow has used zebrafish as a model organism for a series of behavioural/physiological/heritability studies since 2016. The zebrafish used in the present paper, originating from different populations and generations, have been held under slightly different conditions over various studies.

In 2016, a wild-origin population of zebrafish (from Kosi River, India) was obtained. A subset of the original wild-parental generation (WP) was initially used for pilot studies. Then, an offspring generation (WF1) was produced. Detailed information on housing and husbandry of the WP and WF1 fish can be found in Rácz et al. (2019) [73]. For individual identification, all fish were tagged using visible implant elastomer—VIE tags (Northwest Marine Technology, Inc., Anacortes, WA, USA).

In mid-2017, a second population was established consisting of semi-wild adult zebrafish sourced from a fish farm in Singapore. Two subsets of adults were established from this population (FFA1 and FFA2). Another small subset of adults was used for breeding, producing a new in-house bred offspring generation (FF0) and a year later, an F1 generation (FF1). In the fish facility at University of Glasgow, two separate rooms have been dedicated to house the zebrafish populations. The rooms have slightly different features and fish-holding systems. The information about husbandry and environmental conditions is summarized on protocols.io (dx.doi.org/10.17504/protocols.io.bqf9mtr6; Accessed date for study: 1 December 2020). Critical water parameter and husbandry details are provided here in Table 1.

### 2.2. VIE Tagging

The WP fish were initially tagged using instructions in the tagging manual from Northwest Marine Technology, Inc., which was previously adapted for small reef fish [26] evaluated and used on zebrafish [22,25]. Following this protocol (base protocol), we used elastomer + curing agent and MS-222 (Tricaine methanesulfonate) as an anaesthetic compound, without additional steps/compounds. All tagging was conducted in line with advice from the Named Animal Care and Welfare Officers (NACWO) and veterinary team at University of Glasgow.

**Figure 1:**
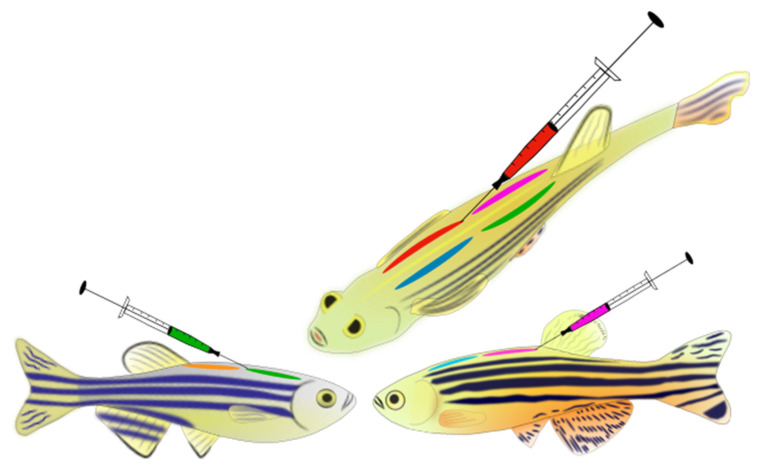
Visible implant elastomer (VIE) tags on zebrafish. Schematic picture of tagged dorsal position of fish in this study (top figure—showing fish dorsal area from above front-left + front-right, back-left + back-right; bottom figures—showing one body side-front and back tagged region on the dorsal area of a ♀ (to the left) and ♂ fish (to the right).

We tagged 400 individual WP fish on four dorsal side locations: front-left, front-right, back-left, and back-right (Figure 1). Five different colours (red, pink, green, yellow, and orange) were used to identify the most visible prominent tags against the skin of the zebrafish. For anaesthesia, we used a one-step anaesthesia set-up, in accordance with general zebrafish anaesthesia guidelines, with the use of 130–140 mg/L dose and a buffered MS-222 solution, which is within recommended ranges [72,74]. Individual fish were placed into a container with buffered anaesthetic solution and system water. Anaesthesia level was evaluated following [45,71], and all fish were anaesthetised to stage 4–5 anaesthesia (i.e., loss of equilibrium and no reaction to stimuli); this took approximately 2–3 min. Fish were then removed from the anaesthetic bath and placed into a wet sponge cut with a groove for tagging. Pre-filled syringes (microfine 0.5 mL insulin syringe mounted with a 29-gauge needle) with suitable VIE colours and curing agents were then used to tag the fish. Fish were lightly held in the wet sponge, and the syringe needle was inserted at a shallow angle under the first layer of the skin on the dorsal side of the fish. Careful effort was made to tag under the dermis, and not into the muscle of the fish. Each fish was tagged four times on their dorsal sides (Figure 1). The tagging process took approximately 2 min per fish, after which we measured mass and length. Following tagging, each fish was placed into an aerated recovery tank. Tagging continued until all fish for that day were tagged (approximately 60 fish). Density in the recovery tank was kept low by introducing new recovery tanks to place fish into through the day. The postoperative care of the fish consisted of 1 h behaviour and health observation/monitoring, followed by an overnight stay in the recovery tanks on a bench in the aquarium room (in a group holding design with 2–3 fish per L density). The following day, fish were observed and sorted, and any fish displaying erratic or abnormal behaviour were closely monitored, and if needed euthanised following laboratory guidelines. All other fish were placed in long-term housing tanks. The recorded mean fork length (FL) of the tagged WP fish was 32.5 ± 2.47 mm (mean ± sd), whilst recorded mean mass of fish was 0.39 ± 0.10 g (mean ± sd).

A second round of tagging was performed on 1100 individuals (WF1) fish. We added a number of refinements to the base protocol. Firstly, to minimise tag loss, tag rigidity was reduced by eliminating the use of the curing agent which is typically used to harden the elastomer. Furthermore, a lower dosage of MS-222 (110–120 mg/L) was used for anaesthesia to facilitate a smoother -recovery and minimise any adverse effects [71]. The remainder of the tagging method and health monitoring stayed as detailed for the base protocol used on (WP) fish. The mean fork length (FL) of the tagged WF1 fish was 23.69 ± 1.938 mm (mean ± sd, max = 30.63 mm; min = 17.92 mm), whilst mass of the fish was 0.238 ± 0.064 g (mean ± sd, max = 0.555 g; min = 0.091 g).

Following the loss of the WP and WF1 populations owing to a combination of disease and mortality incurred due to issues with the base tagging protocol, additional consultation was conducted with the NACWO and the in-house veterinary team. A series of refinements were added following the veterinary panel consultation, and we decided to reduce the anaesthetic levels further, and request approval and adjustment of the project licence to allow the use of sedation instead of complete anaesthesia for tagging (with a dosage of 60–80 mg/L MS-222). At this level the fish did not incur the complete loss of equilibrium but were sufficiently inactive to allow successful tagging. Additional changes to the original protocol also included the use of analgesics and healing agents as after treatment process. A pilot study with 160 adult fish (FFA1) was carried out to quantitatively test this newly established tagging protocol (including the sedation dose of MS-222, no curing agent of VIE, use of analgesic and healing agents). Additional elastomer colours were also used, including white, blue, purple, together with the previously used colours (pink, red, yellow, green). The mean fork length (FL) of the tagged FFA1 fish was 32.60 ± 2.206 mm (mean ± sd) whilst mass of fish was 0.55 ± 0.10 g (mean ± sd).

For the newly established tagging protocol, we continued the health monitoring routine—used previously for the WP and WF1 populations. However, as the healing treatment continued for a 7 day period, the health monitoring of fish in their housing/home tanks was carried out on daily basis until end of treatment, to examine the time scale of tagging site recovery on random individuals from the tagged group.

**Figure 2:**
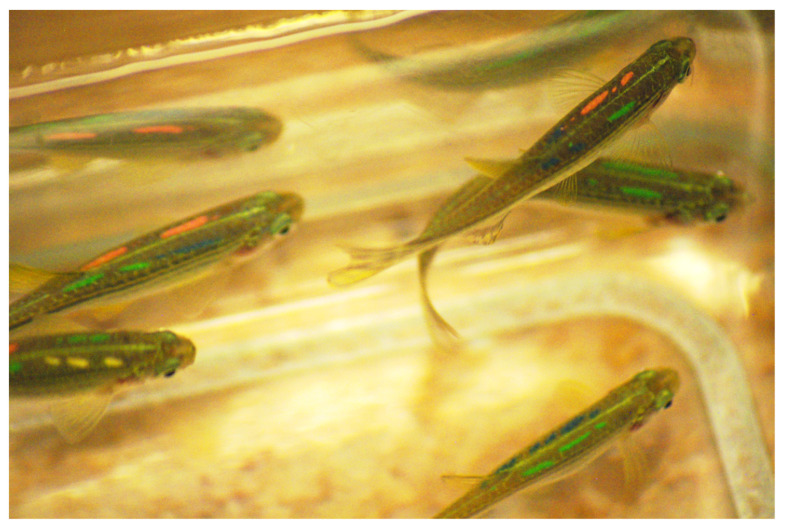
A group of zebrafish tagged with elastomer (VIE) injected beneath the skin. Visible are four coloured marks. Photo credit: Anita Rácz.

Following initial success, a further 790 adult zebrafish (FFA2) were tagged using the newly established VIE tagging protocol. Fish were separated into two populations of 395 fish. In each population, the fish were individually tagged with a unique ID (colour code combination on the four dorsal positions (Figure 1 and Figure 2). Five different colours were used: red, pink, white, green, and blue. For the unique ID codes, an elastomer ID generator software (SalaMarker, developed in the Williams Lab—Purdue University, http://web.ics.purdue.edu/~rodw/salamarker.php; accessed on 12 March 2018) was used. The mean fork length (FL) of the tagged FFA2 fish was 33.523 ± 2.232 mm (mean ± sd) whilst mass of fish was 0.670 ± 0.168 g (mean ± sd).

Upon reaching five months of age, 1800 juvenile size zebrafish (FF0) were also tagged with the newly established VIE tagging protocol. Fish were separated into two populations for tagging. Population 1 consisted of 1080 individuals tagged with a unique four colours code to identify on family and individual level. The same four previously described dorsal positions were used. Population 2 consisted of 720 individuals only tagged to identify on family level. Therefore, only two dorsal positions were used: the front-left and front-right. Six different colours were used (red, pink, white, green, blue, and purple). The mean FL of the tagged FF0 fish was 29.365 ± 2.49 mm (mean ± sd, max = 37.33 mm; min = 16.42 mm), whilst mass of fish was 0.227 ± 0.061 g (mean ± sd).

At five months of age, 750 individuals FF1 were also tagged with the newly established tagging protocol. The set of the study was similar as previously mentioned for the FF0, with two populations. One population consisted of 500 individuals tagged with a unique four colours code to identify both family and individual levels. The same four previously described dorsal positions were used for marking, using the same six different colours. The other population consisted of 250 individuals tagged only for family level on the two top dorsal side. The mean FL of FF1 fish was 28.65 ± 1.88 mm (mean ± sd, max = 39.35 mm; min = 19.13 mm), and the mass of fish was 0.263 ± 0.053 g (mean ± sd, max = 0.607 g; min = 0.129 g).

#### Newly Established Zebrafish VIE Tagging Protocol

Materials required:

- Lidocaine powder (Sigma L7757): Lidocaine stock solution (500 mg/L) prepared freshly weekly with filtered water (or purified reverse osmosis-RO water)(keep the stock solution in a dark or in covered glass bottle at 4 °C)
- Ethyl 3-aminobenzoate methanesulfonate/Tricaine powder (Sigma E10521):

Tricaine (MS-222) stock solution (4 g/L) [74] containing 400 mg of MS-222 in 97.9 mL purified reverse osmosis-RO water; buffered to pH 7.5 with 2.1 mL of 1 M Tris (pH 9) (keep the stock solution at 4 °C for use in 1–2 weeks or at −20 °C for prolonged storage)

- Melafix (API), active ingredient 1%Melaleuca
- Stress & Coat (API), active ingredient 10% Aloe Vera and 1% PVP-I
- VIE tagging equipment (needles, elastomer)
- Sponge fish holder (thick, soft-foam)
- Ethanol min. 70%
- Pre-processing (sedation and analgesia) tank
- Recovery tank (analgesia and treatment tank) with air stone
- Pre-recovery container for weight check (water from the recovery tank)

Preparation for the VIE tagging process:

- Prepare the pre-processing tank: This tank is used for both sedation and analgesia to prepare the fish before the VIE tagging process takes place. Sedation for zebrafish is reached with 60–80 mg/L final concentration of Tricaine (MS-222). Analgesia for zebrafish is reached with 3–5 mg/L final concentration of lidocaine [57,58,66]; (Mocho, J-P personal communication 2017) [75]. Prepare the tank freshly, prior to the VIE tagging process. Use a 3 L fish tank filled with 1 L fish system water.
- Add 7 mL of lidocaine stock solution (500 mg/L), which gives a working concentration of 3.5 mg/L (with a maximum dose of 4–5 mg/L)
- Add ~15–20 mL of Tricaine stock (MS-222, 4 g/L), which results in a working concentration of 60–80 mg/L
- Add air-stone to aerate the tank
- Prepare the equipment for tagging
- Fill needles with coloured elastomers (important: do not use the supplied hardener-curing agent, only use the liquid elastomer ink). Prepare the desired number of colour types and use 70% ethanol to clean all needles before use
- Prepare the recovery tanks: These tanks are used as analgesic and treatment tanks at the same time, to house fish overnight after the VIE tagging process to recover. Prepare the recovery tanks freshly prior to the VIE tagging process. Use larger size fish tanks such as 10 L tanks filled with 8 L fish system water. The size of the recovery tank depends on the number of fish per tank that will be housed overnight. A maximum of 2–3 adult fish per L can be held into the recovery tanks (the number of juveniles or smaller fish per litre can be a bit higher).
- Add 48 mL of lidocaine stock solution (500 mg/L), which gives a working concentration approximately of 3 mg/L (with a maximum dose of 4–5 mg/L)
- Add 1 mL of Melafix (API) as the recommended dose is 5 mL/ 40 L of water
- Add 1 mL of Stress & Coat (API) as the recommended dose is 5 mL/ 40 L of water
- Add air-stone to aerate the tanks.
- Pre-recovery container: This container is used to measure the wet mass and the length of the tagged fish in a less stressful way.
- Fill the container (~50–100 mL) with water from the already prepared recovery tank (to measure the fish wet mass in water)
- Add a small amount of this water to a small plastic zip-lock bag (to measure the fish length while the fish are kept constantly in water in the bag)
- VIE tagging process: Once all equipment for the tagging has been prepared, fish can be separated in their holding tanks. (Feeding should be withheld for 24 h prior the tagging).
- Organise the tagging needles
- Transfer the zebrafish from their holding tank into the pre-process tank to be sedated.
- Once the fish are sedated (approximately 2–3 min, once their swimming slows), remove one fish with a small net or plastic spoon and place it onto the wet fish-holder sponge for the tagging (use water from recovery tank to wet the sponge). Be extremely careful in handling; use rubber gloves to reduce abrasion on the fish and hold the fish in the sponge carefully.
- Tag the fish where needed with the required colours. In this study, four tagging dorsal positions were used (top left, top right, bottom left, bottom right—Figure 1). Carefully eject the elastomer under the epidermis; introduce the needle into the marking location and insert it for around 4–7 mm long. Slowly withdraw the needle while injecting the elastomer. A long narrow mark is created. As it is important that the tag is fully contained under the tissue, avoid extrusion of the material by stopping the ink injection before removing the needle. After injecting all elastomers into the fish, clean any leaked elastomer from the skin of the fish with wet tissue paper. Clean all the used needles with ethanol.
- Transfer the fish into the pre-recovery container and measure its wet mass, then move the fish carefully with a plastic spoon into the prepared plastic bag containing water to measure the fish length.
- Transfer the fish into the recovery tank for treatment and monitor the fish behaviour and full recovery for an additional hour. If a fish looks unhealthy, shows stress symptoms including hyperventilation, freezing, erratic swimming, or any other behaviour which is not in the normal behavioural profile of the species [76], isolate and/or sacrifice it as necessary. After all the fish have been tagged and recovered, leave them in the recovery tank with aeration for overnight.

Post-tagging treatment process:

Conduct further checks on the health and behaviour of the fish the morning following tagging. Healthy and normal behaving fish can be moved into new clean holding tanks in the main system and placed on their normal daily feeding regime. To promote healing and regeneration of any damaged tissue, continue to use of Melafix and Stress & Coat (both at dosage of 5 mL/40 L of system water) for a further six days. In the first 2–3 days use full recommended dose daily, then it can be reduced to half dose daily for the remaining of the healing period.

Melafix and Stress & Coat do not affect the efficacy of biofilters, so it is possible to use them on recirculating systems. Alternatively, fish may be treated in offline tanks, however water quality parameters should be closely monitored, and water changes carried out when needed (on daily basis if possible).

### 2.3. Data Recording and Processing

Tagging mortality rate was calculated as a percentage range, generated from the daily mortalities within each group separately. Tag retention was calculated as a percentage range across groups and included all types of tag loss on individual level, i.e., any fish that lost at least one tag was considered to have experienced tag loss (loss of one, two, or more tags per individual) within a certain measured timeframe (generally over 5–8 months—dependent of study length). All information on injection site healing and disease were made through personal observation from animal care staff. Injection sites were checked daily on individuals for 1–2 weeks (generally until the injection points had healed for the majority of tagged individuals). Ad hoc monitoring of disease occurred over a -1.5-year timeframe. Quantification of tag colour suitability was collated from several group members and partly made through video analysis used in other projects. For post-tagging health and behavioural monitoring, we used personal health check observations on tagged individuals to detect any distressed or abnormal behaviour [76].

## 3. Results

### 3.1. Original VIE Tagging Protocol-Base Protocol

Our attempt at using the original VIE tagging protocol (directly following the instructions stated on the official tagging manual) and in some zebrafish VIE related papers [22,25] resulted in decreased survivorship and health (WP population) (Table 2). Even with the use of a standard recommended dose of MS-222 (130–140 mg/L) for anaesthesia, we observed a high daily mortality rate (20–25%). Mortality seemed to arise primarily as a result of the anaesthesia and not the tagging itself as death, which usually occurred immediately after fish were placed into the recovery tank, within a few minutes or up to maximum an hour following transfer, occurring with gill-bleeding, spiral swimming, and other symptoms after the fish resumed activity. By personal observation, we detected that larger gravid females were more likely to experience mortality within the WP population and died at a higher rate than males. Within the hour of recovery monitoring, most fish remained motionless on the bottom of recovery tank with hyperventilation for approximately 10–15 min, followed by agitated and erratic swimming for additional extra minutes. Tag retention reduced daily and elastomer particles were seen on the bottom of the tanks with the detection of protruding rigid elastomer ends at the tagging site throughout the monitored time frame (two weeks). Furthermore, in the following 5–8 weeks post-tagging, we began to recognize symptoms of disease developing within the tagged group.

After eliminating the use of the curing agent (hardener) for the slightly changed “advanced” base protocol, tag retention rates immediately improved from 60–70% to 85–90% (~100–170 individuals lost some portion of their tags) (Table 2). However, the anaesthetic component of the protocol still proved problematic, as even with a lower MS-222 concentration (110–120 mg/L), the mortality rate was still relatively high (10%, Table 2). All deaths occurred immediately following tagging, affecting the larger gravid females but smaller individuals as well (pers. obs.). During recovery monitoring, we detected similar results as compared to the original base protocol, with fish showing freezing and hyperventilation for approximately 10–15 min post-tagging. However, detection of agitated swimming post-tagging was minimal in this group, and after approximately 15 min, fish started to explore the tank while swimming normally. Skin healing was improved compared to (WP) fish, without protruding elastomer, but skin still healed slowly, taking as long as 7–8 days. Other post-tagging problems still arose within 5–6 weeks, including inflammation and infection. Overall, the tagging procedure was improved but was still not suitable for large-scale tagging operations and experimentation, as we still detected adverse post-tagging responses from the fish.

### 3.2. Newly Established VIE Tagging Protocol for Zebrafish

**Figure 3:**
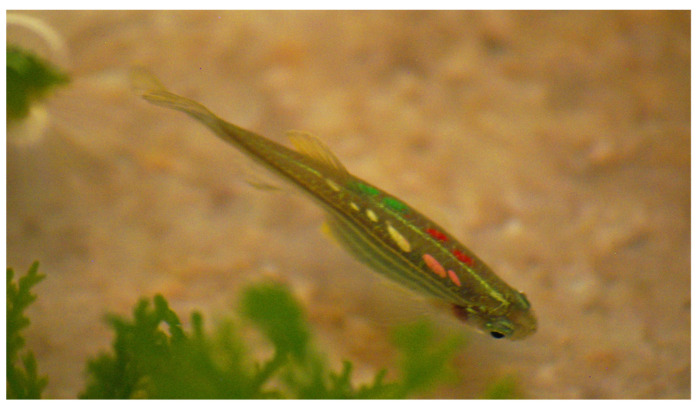
A VIE-tagged individual with fragmented elastomer under the skin. Visible are four coloured marks (as tagged 4 position). Photo credit: Anita Rácz.

Using the newly established VIE tagging protocol during the pilot study (FFA1), we experienced very low mortality following tagging (between 0–2%) (Table 2), losing a maximum of 1–2 individuals from a hundred tagged fish at a time. Post-tagging behavioural observation showed that the use of lidocaine through the tagging process and as an after treatment in the recovery tank helped fish to recover without signs of distress or discomfort. Given that fish were sedated and not fully anaesthetised, they quickly resumed their typical exploratory and swimming behaviours (shoaling within a group) in the recovery tanks. In comparison to fish tagged using the base protocol, fish tagged using the newly established protocol started to explore recovery tanks within approximately 5–8 min without extended periods of motionlessness, hyperventilation, or anxiety-associated behaviours. Following the recommended treatment time and procedures for healing agents, fish fully recovered within 3–4 days. No unexpected deaths or clinical signs of disease occurred during the experimental and holding period (over 1.5 years). Tag retention was almost 100% (only one fish lost a tag in a 6 months period), occasional tag fragmentation occurred over time, but individual fish remained easily identifiable (Table 2; Figure 3).

The marking process of the other group of adult zebrafish (FFA2) resulted in a low mortality rate (between 0–2%, Table 2). Mortalities were typically still constrained to larger gravid female fish immediate after tagging (within 1 h post-tagging), similarly to FFA1, with there being few if any mortalities per tagged group. We saw similar recoveries to fish tagged in FFA1, no recovery issues were apparent and no additional prolongated distressed or erratic behaviour was seen. Overall, no problems or mortality owing to tagging occurred during the full experimental study and breeding period past-tagging (11 months later). Tag retention was high (circa 98–99%) with only around six individuals losing one and around nine individuals losing two tags throughout the study time—within 5–6 months (Table 2). Fragmentation of tags occurred over time, but it remained relatively simple to distinguish between tags, even during video recorded analyses using the fish.

The mortality rate during the tagging process of the in-house bred population FF0 was slightly higher (around 2–4%, Table 2) compared to the adult, FFA1 and FFA2 populations (0–2%), meaning that a loss of 2–4 fish from a hundred tagged individuals occurred at a time. All deaths occurred immediately following tagging and mostly affected the smaller individuals (pers. obs.). Survival rate 24 h post-tagging was 100%, with all mortalities occurring directly after tagging. No additional mortality occurred for the duration of the experimental studies and the breeding period (over 11 months). All tagged individuals which were held for over 2 years have not displayed any clinical signs of disease. Recovery was similar to that observed in the FFA1 and FFA2 populations. No recovery issues were encountered (exploratory and normal swimming behaviour reached within less than 10 min), and injection point healing was as rapid as that encountered in adult fish. Tag retention rate (96–97%, Table 2) was high considering fish were still in a growing period during the tagging. From the 4% (72 individuals) overall tag loss, only 1% (18 individuals) lost two tags, and only three individuals lost more than two tags in long-term from the 1800 tagged individuals. Again, fragmentation of tags occurred over time (Figure 3), but it remained relatively simple to identify the tags, even during studies using behavioural video analyses. When tag fragmentation was high, the use of UV light helped detect small coloured fragments left under the skin.

The mortality rate during the tagging process of FF1 population showed similar results as what we experienced with the FF0 population (around 2–4%, Table 2). All deaths occurred immediately following tagging, and by personal observation, we concluded that in this group, the smaller individuals were again the most affected (with approximately 2–4 individuals experiencing it out of a hundred tagged individuals at a time). As we observed that small individuals may be more sensitive to tagging, it became apparent that fish size (body mass and length) may also be important factors affecting mortality at tagging. All individuals which survived the first hour of post-tagging, stayed alive and healthy afterwards. No additional mortality was observed followed the tagging day and fish remained healthy without any disease symptoms until at least 18 months of age. For recovery monitoring and healing, we detected the same success in the other populations tagged using the newly established protocol. The recovery from the tagging procedure was rapid, followed by a good healing pattern at the tag site. The tag retention rate (98–99%, Table 2) was high, higher than at FF0. Only 1–2% of the individuals (7–15 individuals) lost either 1 or 2 tags in the study period over 5 months post-tagging. Individual identification was easy throughout the study, even during video analyses. Taken together, these positive outcomes suggested that the additional tagging refinements were beneficial to the welfare of our tagged animals.

### 3.3. Evaluation of Colours

Fluorescent colours (red, green, yellow, and pink) and a nonfluorescent colour (white) were easily identifiable on zebrafish by the naked eye and are recommended for use in future studies. However, yellow and white tags are visually similar with the naked eye, and their use is therefore discouraged in the same group of fish population. Confusion between the two can be avoided by using an UV light to read the tags as the yellow colour contains fluorescent properties, whereas the white colour does not. Also, yellow and green can appear quite similar under UV light but are easily recognisable under normal lighting conditions. In addition, some fish when stressed may display darkening of skin, which can lead to difficulty in differentiating particular colours, such as green and blue. Purple (no fluorescence) and blue (fluorescence) are both possible to use if more colour combinations are required. However, they can appear dark against the zebrafish skin and therefore can be difficult to recognise and dissociate with the naked eye, especially if fish are stressed and their skin darkens. Additionally, purple seemed to have a higher fragmentation rate than other colours. Orange was determined to be the least favourable of the used colours, due to its similar appearance to both the red and pink under the skin of zebrafish, potentially causing misidentification if combined its use with either colour. If used by itself, the colour is clearly visible.

## 4. Discussion

Despite VIE tagging being used across a wide range of aquatic taxa [27,28,29,30,31,32,34,35,36,37] a detailed and refined tagging protocol with animal welfare at its core has been lacking. Not only is the elimination of unnecessary distress crucial to a good experimental outcome [47], but it is extremely important from an ethical standpoint as well. Here, we present a detailed tagging protocol, with particular emphasis on the use of analgesics and post-tagging recovery procedures.

We encountered several problems using the base protocol tagging method on our wild-caught fish population (WP, WF1). Some fish exhibited negative responses to the tagging, such as showing oedema (at the site of tags) and signs of infection and disease. Because fish are in direct contact with their aquatic environment, any epithelial tissue damage combined with excess stress can significantly increase the chances of infection through inflammation and immunosuppression [77,78,79]. Previous work on three-spined sticklebacks (*Gasterosteus aculeatus*) has shown that the VIE tagging process including the handling, anaesthesia, and elastomer insertion can stimulate immune response (i.e., increase in granulocyte:lymphocyte ratio—G:L) in the fish [44]. Given that immune suppression is known to lower resistance to disease [78,79,80], it is possible that VIE tagging may have a negative effect on the health of the fish, causing distress and lasting harm. Wild-caught fish may be more prone to stress and have a higher sensitivity to chemical compounds than laboratory bred lines. This may further exacerbate immune responses, leading to lower resistance towards pathogens [78,79,80]. Because of these issues, it is crucial to improve tagging protocols to minimize the likelihood of negative responses from fish, ensuring long term reliability and safety. Using the base protocol, our results contrasted in many ways with previous work evaluating VIE tagging in zebrafish [22]. Hohn and Petrie-Hanson (2013) [22] used the original protocol suggested on 1-to-3-month-old individuals (stated average mass as 0.25 g- and length 3 cm) and observed good tag retention on the dorsal area and no post-tagging effects, such as healing issues or infection. The mortality rate in their study was stated as 0% for all the tested groups. However, it is not clear if they recorded any immediate mortality during the tagging process, or if this value was drawn from individual losses during the time of recovery after procedure. Using the same protocol in our study, we experienced low tag retention as fish frequently lost parts of their tags or even the entire tag, as well as reduced healing at the injection point for weeks with some showing evidence of inflammation or infection [73]. In addition, discussions with other researchers using zebrafish as model species have indicated that similar problems have arisen across different research groups when using the VIE base protocol to mark their laboratory-based lines of zebrafish colony (loss of tags, slow healing, and the start of infections). Therefore, we made several improvements to the base protocol to address the issues. We firstly removed the curing agent from the tagging protocol to make the material softer, hoping that the decreased rigidity would serve to make the tag more flexible and less likely to be ejected and re-open injection sites. Furthermore, we investigated the use of anaesthetic protocols and later an after-treatment to avoid excessive mortality.

Even with great care taken during the tagging process, we experienced some immediate mortality rates directly from tagging (though later it was substantially lower than those experienced through the base protocol). It is possible that these higher mortality rates may have been due to the dosages of anaesthetic used (140 mg/L MS-222). Tricaine methanesulfonate is typically considered a safe anaesthetic for fish [45], which is a commonly used anaesthetic and euthanizing agent across the zebrafish research community [12]. However, in some cases, it is also known to cause side effects, such as changes to the cardiovascular and endocrine systems as well as to osmoregulation and ion regulation [81], which may cause hypoxemia, hypercapnia, respiratory acidosis, and hyperglycaemia in fish [41]. It also has been shown that MS-222 can have negative effects on zebrafish specifically, depending on the dosing, the exposure time, and on individual sensitivity (different sexes and age groups may react differently) [39,40,72]. Alternative anaesthetic compounds as a replacement for MS-222 are still under evaluation [39,40,46,82]. In the meantime, it has been advised to investigate and refine anaesthetics protocols for the fish [59]. Our investigation and trials helped us to choose to use a safer/lower dosing level of anaesthetic. After veterinarian consultation and license approval, we decided to allow the use of a sedation level (~60–80 mg/L MS-222) for the VIE tagging process, as opposed to using a typical level of anaesthesia adopted for surgical procedures (i.e., level where total loss of equilibrium is experienced). This level of anaesthesia helped to hasten fish recovery and maximise survival.

Further improvement of the VIE tagging protocol required the consideration of important fish welfare questions. For example, the question of whether or not fish feel pain and experience distress has been explored for over a decade, and there is evidence to support the idea that fish do indeed perceive pain. The ability to react to potentially damaging stimuli is seen across numerous taxa and is mediated through nociception [59,62]. Nociception represents the basic mechanism for the detection of pain [66] through nociceptors: receptors that preferentially detect harming stimuli [59]. Previous research revealed that fish nociceptors react to a variety of stimuli, including chemical, physical, and other environmental stressors, similar to those of humans [52,53,54,55,58,59,61,62,83].

The refinement of existing scientific protocols to reduce invasiveness and minimise potential pain to animals is central to the 3Rs principle [84], which aims to provide reliable scientific outcomes, as well as avoid unwanted immune reactions, infections, lasting harm, and unnecessary stress. This has encouraged researchers to acknowledge that fish models should be handled more carefully during scientific procedures and has fuelled the need for further investigation in the field to evaluate analgesia for the aquatics species as well [61]. Several recent studies have investigated the impact of analgesic drugs in fish with noxious stimuli [57,58,63,64,65,66]. Work has shown lidocaine to be an effective analgesic drug, reducing the effects of potentially painful stimuli in both juvenile and adult zebrafish [57,58,64,65,66]. Given the importance of reducing stress and the elimination of long-lasting harm in scientific procedures [47,58,84], we decided to include lidocaine as analgesic into our newly established tagging protocol. We also further improved the protocol by include the widely used hobby-aquarist healing agents Melafix (active ingredient 1% Melaleuca) and Stress & Coat (active ingredients 10% Aloe Vera and 1% PVP-I) to increase the healing process after the caused tissue damage in the tagging method. Because we used a sedation dose of anaesthesia together and followed by analgesia, fish returned to their normal “standard” behaviour (first exploration, followed by their normal shoaling swimming) both more quickly and smoothly than without it. As with the use of Melafix and Stress & Coat, the puncture spot-marks left by the needle healed completely within a few days’ time post-tagging, and the fish stayed healed and healthy for the study duration (in some cases over 1.5 years).

Due to differences in husbandry and rearing methods among experimental facilities (e.g., feeding regime and densities), growth rates between populations of fish are likely to vary significantly. Therefore, we suggest that size as opposed to age should be used as a proxy for when fish are ready to be tagged. Given the small general size of zebrafish, and their seemingly high sensitivity to tagging, we would suggest to avoid tagging juvenile zebrafish below 22 mm standard length with an approximate minimum of 0.200 g mass with VIE. Size-dependent mortality has been observed with other fish species, where younger and/or smaller individuals have had higher mortality with VIE tagging [26,85]. However, some other studies have not identified any significant issues using standard tagging protocols on juvenile or small-sized fish [22,86], or have detected differences between the cured or uncured tagged groups [87]. This suggest that sensitivity to tagging and tagging success can vary on a population-by-population and species-by-species basis as well. Therefore, we recommend that individual studies should choose tagging protocols that are most suitable for their objectives and evaluate them inhouse before carrying out major tagging projects.

Observations of VIE colours indicated that all fluorescent colours (red, green, yellow, pink, orange, and blue) and nonfluorescent colours (white and purple) are visible under zebrafish skin. When a single colour is being used, we suggest that all of these colours are suitable for zebrafish. However, when several colours are needed, attention needs to be taken in which are chosen to be combined. Green, yellow, pink, red, and blue are suitable together as they can easily be recognised from one another. We discourage the use of white-yellow, orange-red, and orange-pink combinations, as these are nearly identical to the naked-eye. In situations where the skin tone of fish is dark, green, blue, and purple can also prove difficult to discern. Researchers should choose the most appropriate colour based on the tone of their fish, as well as the purpose of the study.

The current study was opportunistic in nature and was therefore not hypothesis driven. Despite this, we believe that this information will be extremely valuable for researchers aiming to use visible implant elastomer with zebrafish or other small fishes, by maximising animal welfare and the value of collected data. The information herein can also be used to design more directed, hypothesis-oriented studies, for further refinement of tagging methods.

## 5. Conclusions

Until now, the zebrafish research field has lacked a dedicated and well-described individual identification marking method with animal welfare and the 3Rs at its core [88]. The newly established VIE tagging protocol described in the present study appears to be a reliable and safe method to use on zebrafish. This protocol could be beneficial to the zebrafish research community and beyond by providing aid where reliable marking techniques are required to identify individuals or genetically diverse lines (mutant and transgenic). The use of this marking method on zebrafish will make it safer to house and breed different lines together, minimising the potential of mixing genetically diverse groups. The proposed protocol also has potential to be used with other fish species with slight amendments where needed, and could also be used to improve refinement and animal welfare for other marking methods and/or nociceptive procedures, such as scale removal or the commonly used spine- or fin-clipping methods.

## Figures and Tables

**Table 1:** Summary of the water quality parameters and husbandry routine in the facility.

Environmental Conditions	Room 1 Z-Hab Unit	Room 1 300 L Glass Tanks	Room 2 Rearing Unit + Glass System
Water temperature	26.0–28.5 °C	26.5–27.5 °C	26.0–28.5 °C
pH	7.0–7.5	7.0–7.5	7.0–7.5
Conductivity	~400 µS	~200 µS	~200 µS
NO_2_	<0.05 mg/L	<0.04 mg/L	<0.06 mg/L
NO_3_	<15 mg/L	<10 mg/L	<15 mg/L
Ammonia	<<0.05 mg/L	<<0.05 mg/L	<<0.05 mg/L
Fish feeding <4 months	4× daily	ND	4× daily
Fish-feeding regime > 4 months	2× daily	2× daily	2× daily
Tank cleaning regime larvae–juvenile	<1 month–daily; >1 month–weekly	<1 month–daily; >1 month–weekly	<1 month–daily; >1 month–weekly
Tank cleaning regime Adults	Monthly (wash tanks)	3–4 weekly (clean and siphon)	3–4 weekly (clean and siphon)
Adult fish-holding density	5 fish/L	1–2 fish/L	3–6 fish/L
Light cycle	13 h L: 11 h D	13 h L: 11 h D	13 h L: 11 h D

ND: no data.

**Table 2:** Summary of the mortality (health status) and VIE tag retention with the original-base tagging protocol and after with the newly established tagging protocol has been used on the different fish populations. Percentages for mortality and tag retention rates are ranges from data collected across groups of fish tagged on different days. Separately coloured shades used in table highlight the separate tagging methods.

Group of Fish	Injection Point Healing	Tagging Mortality Rate	VIE Tag Retention	DiseaseAfter Tagging	Number of Tagged Fish	Tagging Method
Wild-origin parental population (WP)	long periods	20–25%	60–70% retained	YES	400	base protocol
F1 generation of wild population (WF1)	over a week	10%	85–90% retained	YES	1100	advanced base protocol
Fish-farm-origin adult population pilot (FFA1)	a few days	0–2%	99–100% retained	NO	160	newly established protocol
Fish-farm-origin adult population (FFA2)	a few days	0–2%	98–99% retained	NO	790	newly established protocol
Fish-farm origin in house bred new generation (FF0)	a few days	2–4%	96–97% retained	NO	1800	newly established protocol
Offspring of the in house bred FF0 population (FF1)	a few days	2–4%	98–99% retained	NO	750	newly established protocol

## Data Availability

Not applicable.
